# Making with the Trouble: Un/Enfolding Posthuman Participants with Young People in Creative Post‐Qualitative Research

**DOI:** 10.1111/jade.12580

**Published:** 2025-05-08

**Authors:** EJ Renold

**Affiliations:** ^1^ School of Social Sciences Cardiff University Cardiff UK

**Keywords:** creative methods, new materialism, participatory, postqualitative, sexuality education, visual arts, young people

## Abstract

This paper explores how ‘what matters’ can surface in multisensory arts‐informed projects as ways for young people to survive and stay with gender and sexuality troubles that are always more than theirs. Situated in an ex‐mining post‐industrial locale, we make an agential cut in a longitudinal research and engagement project called Unboxing Relationships and Sexuality Education (RSE) to open up a rare case study of entangled ‘creative coproduction’ (Renold and Ivinson forthcoming). Supported by an artist‐in‐residence teacher assistant, composer and filmmaker, we explore the making and mattering of a clay sculpture, the Bruised HeART, created by Alys (pseudonym, age 13) which we theorise as a ‘dartaphact’ (a concept combining ‘data’, ‘art’ and ‘act/ivism’ to register the posthuman participation of arts‐based data). We follow how the heART continues to matter through film and a second dartaphact made from barbed wire and skewered fragments of Alys' instapoetry. Drawing upon the concept of The Fold we compose three figurative folds (crushing matters; crystalising matters; carrying matters) to draw connections between Alys' activist mining ancestors, the silencing of queer violence, and her collection of locally sourced crystals – situated practices generating resourceful posthuman companions to manage multiple troubles. Each fold is composed for its passageway potential to glimpse at our ethical‐political praxis of attuning to and making‐with troubles already in motion and how dartaphacts might propel new ways of understanding and doing relationships and sexuality education Otherwise.

## Introduction: unboxing methodological matters

We return to the art classroom in a secondary school in a Welsh ex‐mining community where we have worked for many years. We curate the space to explore the power of art to unbox what matters for young people as part of a participatory relationships and sexuality education research and engagement project. Young people's portraits stare down from the ceiling, multi‐coloured papier‐mâché monsters line the windowsills and fragments of past artworks wait patiently for what next they might become. Alys (pseudonym, age 13) takes up the challenge. She takes out the sticky‐note messages in her glass jar that capture ‘what jars’ her most about relationships, sex and sexuality (Figure 1). She starts to mould clay into the shape of a heart. Each sticky‐note is tightly rolled up and inserted into the heart. She stretches, elongates, and smooths another piece of clay into what appears to be a tongue. She lifts the piece of clay and grafts it onto the middle of the heart. We detect a focused fury that fuels multiple further creative cuts. The heart is stabbed with scissors and the tongue is snipped, screwed and stapled to capture, “the silence and torture of how society won't listen”. Under the tongue, the words: ‘rape, racism, homophobia and transphobia’, are written (Figure 2).

**Figure 1:**
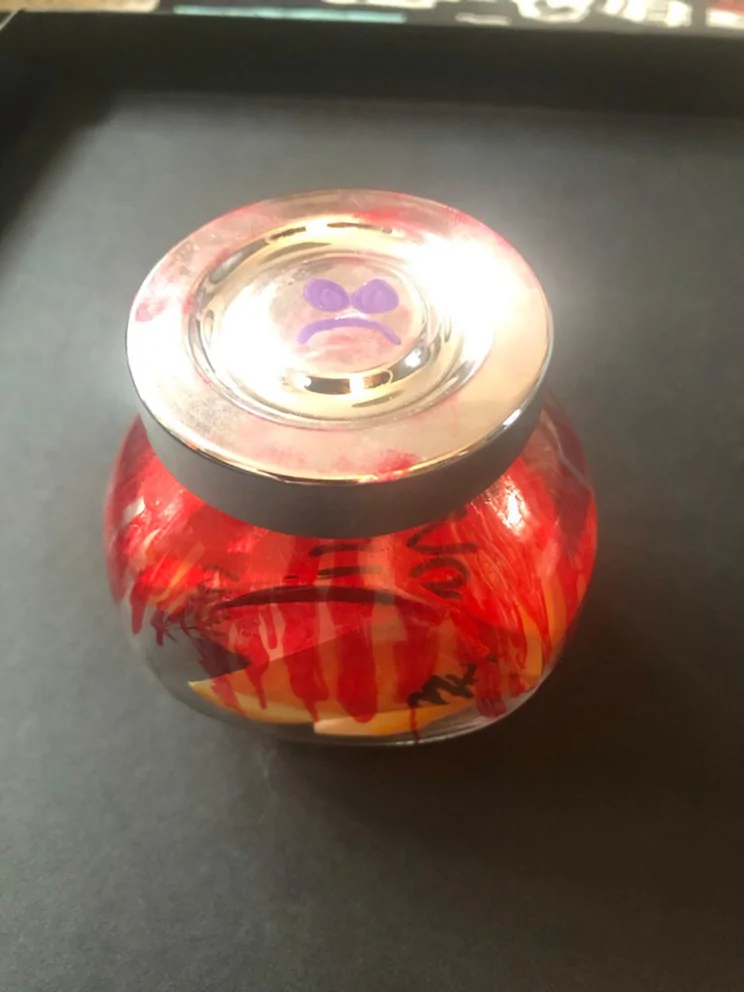
What Jars You? A red‐tinted small glass jar filled with yellow sticky notes. The silver lid has a face drawn on it, with purple eyes and a downturned mouth.

**Figure 2:**
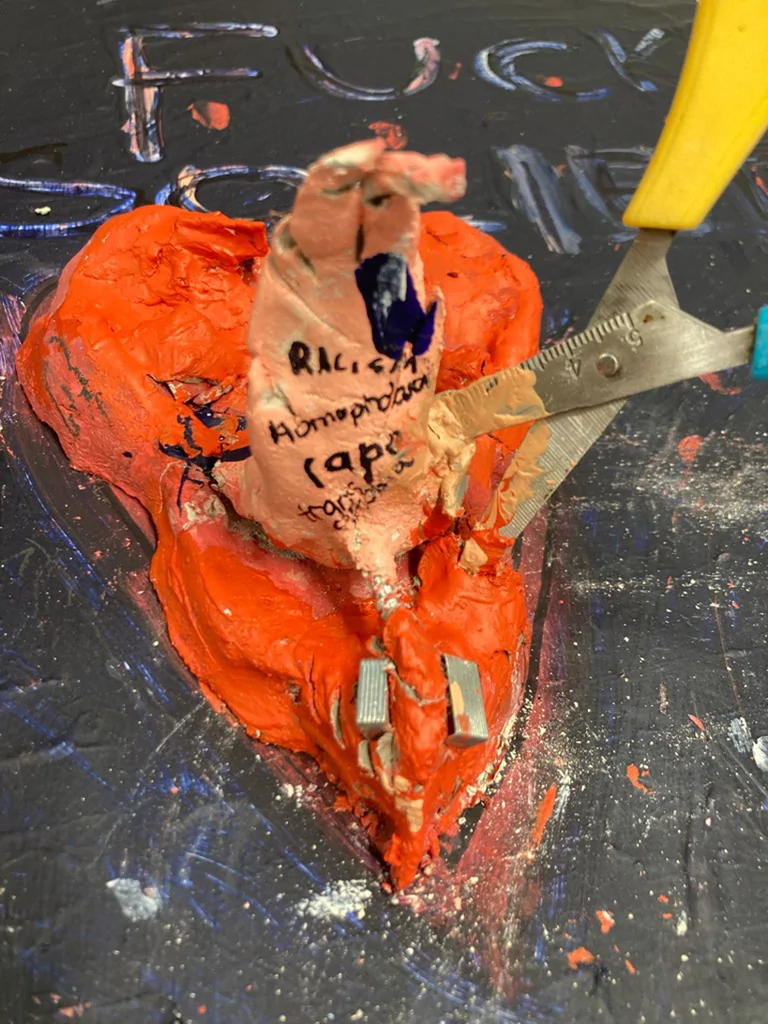
Tortured HeART. A red‐painted clay heart with scissors inserted into it. A long, curved clay tongue extends from the heart. Beneath the tongue, the words ‘racism’, ‘homophobia’, ‘rape’ and ‘transphobia’ are written in black ink.

In the months that follow, Alys takes up the opportunity to work with two artists to animate her heart with sound and movement. She selects an instapoem from an unknown source on her Instagram feed: “In the darkest nights, come with me to the edge of the whispering woods. Across the bridge and over the still river you'll find me there waiting”. She invites our film‐maker to “be inspired by the quote” and for our composer, to intersperse “muffled voices” with young children's “cries that get louder and louder” amidst the sound of a “beating heart”. The artists respond and a two‐minute film is created and shared for comment.

The film (https://vimeo.com/828991152) opens with the sound of a beating heart and the heart slowly comes into view. The lines of the poem score the frames that follow. The heart lurks at the back of a dark forest, accompanied by a baby's cry and indecipherable chatter. The tongue begins to move to the movement of a rippling river, and the volume of the beating heart, cries, and chatter increases. The forest comes into view and three tongued hearts hang on the thorns of the trees alight with fiery embers. The final scene cuts to the heart on fire, slowly burning to the sounds of post‐industrial hammering (Figure 3). The heartbeat fades away. The forest returns, backlit by a shard of light.

**Figure 3:**
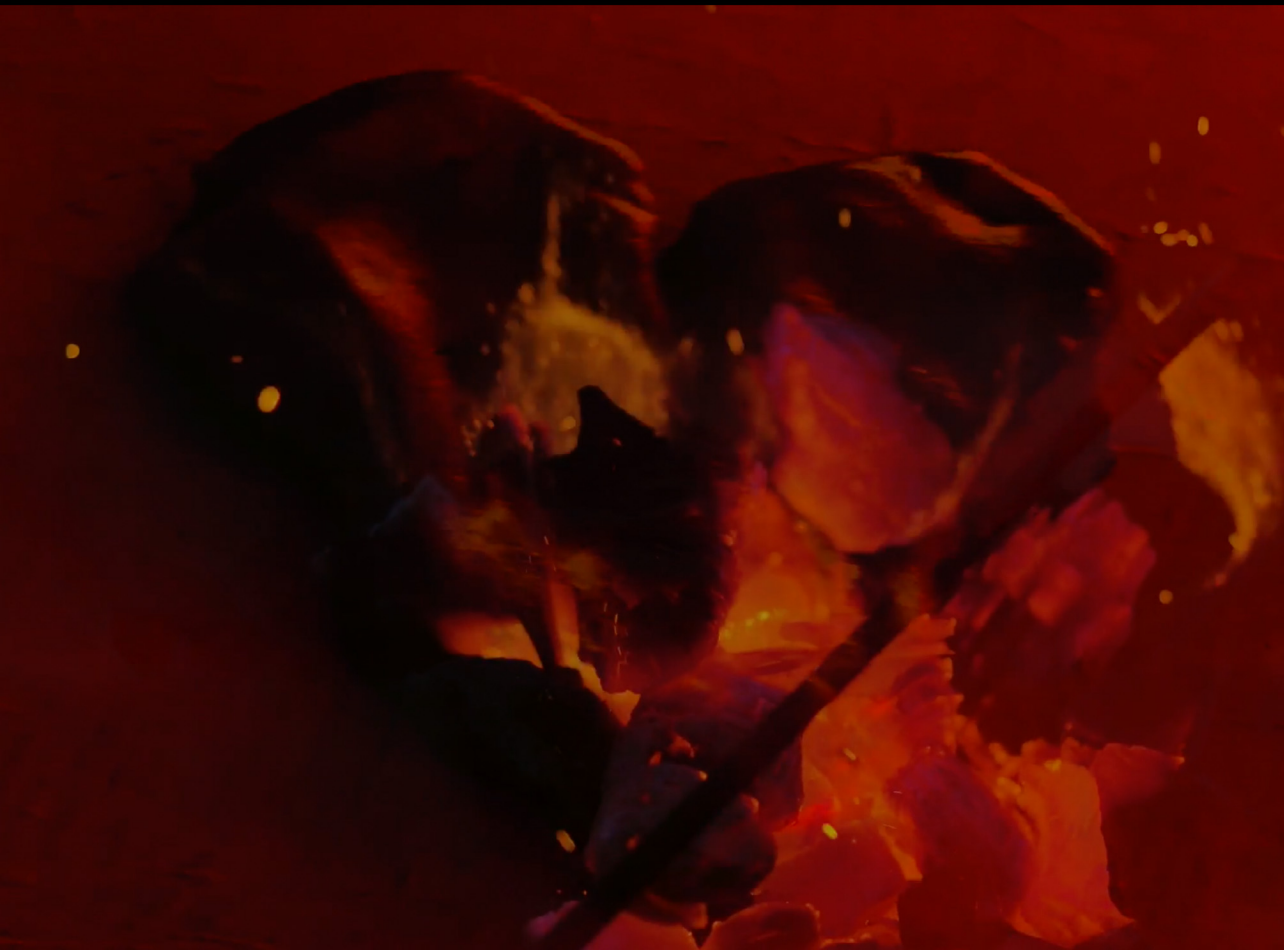
Forged HeART. A digital image of a blackened heart bursting into flames. A faint outline of a blacksmiths hammer is superimposed onto the image.

On viewing the final film, Alys whispers: “it's like she's (the artist) crawled into my mind”. The making of the Bruised HeART is dense with folded relations, but none of us quite know this yet.

The making of the Bruised HeART sculpture and film occurred during a longitudinal participatory research and engagement project^1^ called Unboxing Relationships and Sexuality Education (RSE). The project^2^ builds upon an arts‐praxis of slow creative coproduction in a secondary school art classroom in an ex‐mining post‐industrial locale in the south Wales valleys (Ivinson and Renold 2020). Supported by an artist‐in‐residence teaching assistant, (Sior, pseudonym), a composer (Rowan) and a filmmaker (Heloise), the project worked with a diverse group of 20+ young people (ages 13–14) to creatively explore what might be mattering to them in the broad field of RSE as set out in Wales' new mandatory RSE curriculum (Welsh Government 2022a, 2022b). This is a new rights and equity based, cross‐curricular and LGBTQ+ inclusive RSE curriculum where teachers are expected to co‐construct the broad ‘what matters’ learning objectives with children and young people. It is also a curriculum where the Expressive Arts are explicitly acknowledged as a ‘powerful medium to explore and express feelings, thoughts, views and experiences on a range of relationships and sexuality education (RSE) issues’ (ibid) although practical examples are scarce. Central to our creative approach is an ethical and political responsibility to notice and call out practices that subjugate, silence, sensationalise, silo and simplify children and young people's experiences and to ‘stay with the trouble’ that arises (Haraway 2016).

The jar that features in the opening vignette comes from an activity from the first session^3^ of the Unboxing project (see Renold and Ringrose 2019). It is one of six activities where we invite young people in small friendship groups, to participate in a two‐hour ‘creative audit’ focused on what and how they want to learn about RSE (for details see Renold and Timperley 2024). Following this, young people had the option to continue working with us to create their own artefacts (individually or in groups), or what we theorise as ‘dartaphacts’ (a concept combining ‘data’, ‘art’ and ‘act/ivism’ to register the materialist posthuman participation and activist agency of arts‐based data, see Renold 2018), with the potential to be shared with others (e.g. other young people, teachers, professionals, policymakers and integrated into professional learning resources). This second phase included the opportunity to reanimate their creations through sound and film with artists Heloise and Rowan, whom we have collaborated with for over 9 years.

Eleven young people participated in this second phase, which included over ten additional workshops spanning 12 months. Over the weeks, the art room became what Manning (2009, p.30) describes as a relational ‘contact zone’ where young people began ‘improvising with the already‐felts’. Matters which started as a message in a jar soon became much more (see Renold and Timperley 2024). A living rhizomatic curriculum (Snaza and Mishra Tarc 2019) was in full swing, creatively unboxing a range of RSE concepts, from emotion, consent and human rights to LGBTQ+ identities and sexual violence (see the film, ‘Becoming artful with RSE’). Collectively, they seemed to powerfully enact the civic/activist potential (Thomson and Hall 2020) in one of the core ‘what matters’ RSE curriculum objectives: ‘An ability to advocate for and advance the rights of all and understand and respect all people in relation to sex, gender and sexuality’ (Welsh Government 2022a). We continue to work with young people for an additional year (2022–2023) as their dartaphacts make their way out into the world, during which time we conduct a series of post‐project intra‐views^4^. Like many longitudinal co‐produced projects that build in a research capacity to follow what might come to matter, we extend the project to continue working further with one group and one young person, Alys (age 13) for another school year (2023–2024). It is the unanticipated making and mattering of Alys'^5^ sculpture and film featured in the vignette above that focuses this paper.

## Folding with a post‐qualitative arts‐praxis

The focus on ‘unboxing’ originally emerged as a practical idea to bring boxes of creative audit materials into the art classroom. However, it also resonated powerfully with us in terms of the project's speculative praxis towards art as a passageway of coming to think‐know‐feel differently (Manning 2020). Our approach is not about utilising arts‐informed methodologies for ‘data’ waiting to be collected or seeking to define art's value in advance. Rather, it involves working creatively with a ‘problem space’ (Lury 2020) already mattering and drawing on ‘art's processual capacity to foreground passage and make felt’ (Manning 2020, 22). Its value lies in its force for onward communication and an ethical engagement with the qualitative multiplicity of what might come to matter in the process.

Our creative co‐productive praxis resonates with the turn towards arts‐based new‐materialist post‐qualitative inquiry for social change (see Rosiek 2021; Rosiek *et al*. 2024). For us, this involves how attuning to, sharing and acting upon ‘what matters’ emerges from a careful, ethical–political practice of being and becoming‐with others (e.g., people, places and objects). For scholars new to the ethico‐political ontologies of doing entangled research, this praxis challenges not only what counts as research but also how the methodologies that form our research‐creations (Truman 2021) with young people matter in the world (Taylor *et al*. 2020; Jackson and Mazzei 2022). This means paying close attention to the embodied and embedded nature of doing ‘research’, where research ‘data’ are simultaneously personal, performative and full of transformative potential. Like many post‐qualitative arts‐informed youth researchers, the art in our praxis is thus processual, relational and potentially change‐making (for example, see Coleman *et al*. 2019; Hickey‐Moody *et al*. 2021; Adams and Gannon 2023; Pihkala and Huuki 2023). At the heart of this paper is the formation and transformation of how creative methods make ‘what matters’ with and for young people and how our methodologies might cultivate a living ethics for becoming ‘response‐able’ (Barad 2007) with what unfolds.

Marks' (2024) ontological re‐working of the folded cosmos forms the theoretical foundation of this paper. Mining the swirl of process philosophies and their indebtedness to the relational ontologies of Indigenous knowledge‐practices, Marks makes several moves which we have synthesised, albeit loosely, for this paper to locate our orientation to how creative methods make ‘what matters’.

In a processual cosmology, every being, from a particle to a plant to a person, and accordingly, a dartaphact (as posthuman participant), is alive. Each living being has experience that is received from and acts upon the world. As Marks succinctly sums up, ‘experience expresses us, not the other way around’ (2024, p.11). The cosmos (i.e., the world we are already a part of) flows through us and into all beings. Following Bergson, Pierce, Whitehead, Simondon, Bohm, Deleuze and Glissant, Marks describes this flow as a folded cosmos that operates along two poles:

At one pole, everything enfolds into a teeming unity. At the other pole, everything is constantly unfolding, each according to its own manner (Marks 2024, p.9).

In this dynamic cosmology, all living beings (i.e., anything that feels, acts and communicates) are microcosms living in the temporary folds that we share with others. Constantly in motion and pliable, and thus always open to the unpredictability of life, the fold is like a never‐ending pleated skirt, significantly carrying the micro‐activist cosmos of collective action. Importantly for this paper, change and transformation come from within this pleated milieu (the folded cosmos) and are always local and ‘adaptive to specific local conditions’.

In dialogue with post‐qualitative researchers who are exploring the political potential of creative methodologies to attune to the folds at work in specific events or situated practices, in this paper, we focus upon a rare moment of deep entanglement in the making and mattering of the Bruised heART – a dartaphact dense with folded relations. Via a diffractive analysis (Lenz‐Taguchi and Palmer^2013^, Bozalek and Zembylas 2017, Mazzei 2014), that allows us to detect fragments of an unfolding (future‐oriented) and enfolding (protective) cosmology, we open up some of the folded pleats and their haptic relationship to time and space. To retain the speculative aesthetics of this praxis, the paper is structured around three figurative folds. These folds are not chosen at random. Rather, as Marks (2024, p. 92) advocates, they have been selected for their productive capacity in how they already ‘hang together and do something’ much like the posthuman agency of a Deleuzian assemblage. We draw upon Haraway's notion of ‘figuring’ to capture the creative co‐productive practice and process of ‘becoming‐with each other in surprising relay’ (Haraway 2016, p. 3). Each fold is figured as a series of matters (Crushing Matters; Crystalizing Matters and Carrying Matters), composed for their passageway potential and mattering within and beyond the Unboxing RSE project.

## Crushing matters

For many young people in the project, the meaning of what matters often came through in the act of making (Manning and Massumi 2014). It wasn't until our post‐project intra‐view, months later, that we became aware of how Alys' intention to sculpt a ‘cute’ heart‐shaped rainbow, inspired by the LGBTQ+ TV series ‘Heart Stoppers’, was rerouted as the project's arts‐activist emphasis was progressively realised:

Alys: Originally … I was going to paint a heart rainbow … but then I realized. Hmmm. And then that is when I put the tongue on. And that is when, you know, I put scissors in the tongue (…) like, not a normal tongue, a curved kind of tongue (Figure 4)

**Figure 4:**
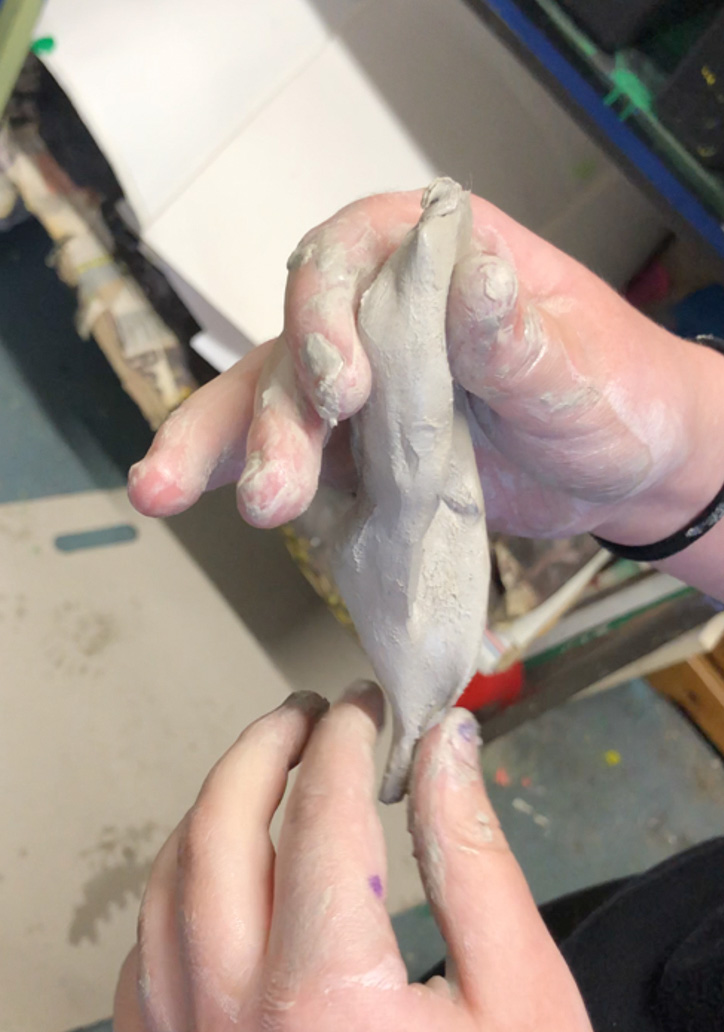
Curved Tongue. Two hands are smoothing out a strip of clay.

Dripping with political potential in its clarion call to ‘f*ck society’, with its endemic ‘racism’, ‘homophobia’, ‘transphobia’ and ‘rape’ culture, the Bruised heART became a dartaphact that seemed to materialise a visceral, organised queer r/age^6^, where developmental age‐appropriate discourses (Robinson 2013) deem Alys, age 11, as ‘too young to be gay’, ‘too young to know’ and speak out about ‘society's disgusting standards’:

Alys: Heterosexuality is the standard, like white is the standard, and like, you know, like being your biological sex is the standard, being weak as a woman is the standard … it's disgusting

Pre‐teen Alys called out these standards through multiple social media platforms:

Alys: I'm that activist … like on Reddit, and Instagram, and TikTok and stuff (…) I'm just like‐ I'd be trying to change their minds, …. I'm sick of being silenced. So, I was like, ‘What can I do to put my words of how I feel out there?’ So, like when I was making the tongue, I realized … like they don't let me talk, they – like they won't let me. So, I was like making a damaged tongue because that's what they've done to me, like they've stopped me from talking.

She explained that the curved tongue, inspired by the ‘Attack on Titan’ Japanese manga series, was based on the backlash she had received for making trouble as a young digital feminist activist, and for speaking openly and being out about her sexual identity in primary school. We unfold this connection a little further.

Attack on Titan is located in a world where humanity is forced to live in cities surrounded by three colossal walls that protect inhabitants from huge man‐eating humanoids known as Titans. In this dystopian world, humanity is the monstrous in/human other, and venturing beyond the city walls is strongly discouraged. The Titans have posthuman strength where body parts, much like the spliced organs of the clay‐heart‐tongue, can harden and become impenetrable. Multiple affective resonances surface between Alys' journey and the series Titan Attacks. Alys has ventured to society's ‘outside.’ She reflects on being an ‘outsider’ in her school, in her community and online from a young age. When we meet Alys at the beginning of the project, she had very much retreated into herself, but in the making of the heart, we perhaps glimpse a posthuman strength enfold (protected) and unfold (become more‐than).

Over the weeks, Alys sculpts a suppressed queer r/age that had increasingly been difficult to communicate. She later talked about how the Unboxing project became an energising lifeline, ‘helping me with my mental health’. She looked forward to the sessions in the art classroom, which were sometimes, she says, ‘the only reason I came to school’. The workshops and the space seemed to give her a sense of belonging (Thomson and Hall 2021), enabling her to relax and create without fear of judgement or censorship – a making with the trouble, that we explore further below to glimpse at the unfolding/enfolding aesthetics of ‘something emerging within and beyond you’ (Marks 2024, p.57).

We find out long after the film had been made that Alys has a strong relationship with feminist and queer instapoetry (Knox *et al*. 2023), especially the poets Rupi Kaur, Atticus, Amanda Lovelace and Ariana Reines. She started writing her own instapoetry in Year 7 (aged 11) and even shared some in the intra‐view – a haunting and uncanny moment where the poem she selects depicts thorns and withered, pierced hearts (written months before the making of the film which includes the same imagery).

You are a garden of thorns
Approaching you is peril in itself
But you welcome me in with open arms
And caress me
With your sharp thorns
You scrape my skin
But little did I know
You're attempting to reach deep within
To prick my withered heart
(Unspoken^7^, age 14)

Marks writes about how ‘the heart of every subject pulses with relations’ (2024, p.53) where ‘the unfolding can make you tremble when something is too difficult to bear because it makes itself felt’ (2024, p.93). In making the Bruised Heart film, these relations were immediate and gut‐felt. The film seemed to open up a creative interval and ignite a wider assemblage, allowing us to experience the haptic touch of the crushing ‘tips and folds’ (ibid) vibrating throughout the making process.

As we are watching the film, Alys reflects on the heart bursting into flames and talks about how the forces of suppressed emotions can burn and explode: ‘It's like you've been through so much and then you're just – you explode … and then, like, it's on fire.’ As Marks (2024) comments, ‘the more relations an entity sustains, the more intense it is’. However, the flaming heart at the end of the film hooks into a wider assemblage – a folded cosmos of transversal connections to the activist legacy of Alys' mining ancestors and a feminist and queer resurgence of breaking the silencing of community violence.

The sounds and images in the film (https://vimeo.com/828991152) are reminiscent of the hammering, flames, seismic ruptures and molten gold, relating to the industrial landscape that this town and the South Wales valleys more widely were at the epicentre (see Ivinson and Renold 2020). We mention these resonances to Alys to see if our attunement to the industrial heritage of place connects with her in some way. Remarkably, it does. Alys tells us that her great‐grandfather had pulled children from the slag heap that had engulfed a primary school during the Aberfan disaster in 1966. The local miners had been telling the mine owners for a long time that the land was unstable and repeatedly warned of a disaster waiting to happen but were not listened to. Women were also speaking out. LGBTQ+ historian Daryl Leeworthy (2017) notes that more than 2 years earlier, at a meeting of the town's planning committee, Councillor Gwyneth Williams had warned that ‘we have a lot of trouble from slurry causing flooding. If the tip moves it could threaten the whole school’. Following the disaster, Leeworthy explains how ‘bereaved mothers met regularly on Tuesday nights at the Aberfan Hotel to discuss what had happened, to talk through their grief and suffering, to cry’ (ibid). These gatherings sparked a renewed impetus, from exercise classes, playgroups and a series of public talks, including ‘a doctor from India who talked about the changing roles of women on the sub‐continent’ (ibid). Forged from the ‘cries that matter’ (Stengers 2021) new alliances with women across the South Wales Coalfield were forming in ways that opened up a diverse support base (including the London‐based Lesbians and Gays Support the Miners). Leeworthy (ibid) speculates that these alliances ‘did so much to encourage the wider labour movement to take LGBT rights seriously’ that ‘it is entirely possible that attitudes towards LGBT citizens would not be as embracing today’.

In a speculative ethical–political arts‐praxis, it is sometimes possible to follow what flows in ways that allow researchers to glimpse an ‘entanglement when an event distant in time and space flashes up and reveals its involvement in the here and now’ (Marks 2024, p.33). Drawing on Deleuze, Marks (2024, p.76) writes about this process as a form of ‘involution’: ‘to involute is to make a fold that connects with allies in other times and places’. We speculate that in the making and mattering of the Bruised heART (as sculpture and film), these latent connections bubble up; an assemblage of ‘crushing matters’, that surface as a transversal field of territorialising and deterritorialising forces, folds and feelings. We speculate that the unfolding/enfolding of women's activism emerging within and from the Aberfan disaster connects not only to Alys' present experience of not being listened to and persistently silenced on matters of gender, sexual and racist violence, but also, connects to an activist community beat – a beat that continues in Alys' digital activist practices vis‐à‐vis her own and others' instapoetry. We could understand these connections as a folded creative legacy of local and global knowing, learning and change‐making propelling a queer r/age to ‘change minds’ and strive for a better world that is hers and more than hers.

## Crystalising matters

Following the project's launch that we co‐produced with young people at the Welsh Government‐sponsored Pierhead Building (see Renold and Timperley 2024), Alys and one other group were keen to continue to work with how else their artworks and the process of making, might continue to matter. For Alys, the *Unboxing RSE* project appeared to spark a growing awareness of how, in her words, ‘if you can't say it out loud, you can say it with art’. Having never previously considered GCSE^8^ Art as an option, Alys decided to take it as one of her subjects. She expressed to us a curiosity about further exploring connections between her own poetry, her clay heart‐tongue piece, the co‐produced film and the visual arts more broadly. In discussion with Sior (teaching assistant), we developed a process that allowed me to access some of the timetabled art lessons and document some of Alys' GCSE art journey (including audio‐recorded verbal reflections, and anonymous photographs and video footage of the making process). This became a generative process that aligned with the requirement for students to reflexively document what and how they are learning and how this learning informs their creative process.

In our first meeting of the new school year, I bring in the photobook which visually depicts the making of the Bruised HeART and Alys brings in a poem about her ‘heart breaking into shatters’ during a previous relationship break‐up. The last four lines read:

I could never understand what was so illuminating about you to me.

But now I realise it wasn't the way you looked or the way you sounded.

It was the way you made me feel.

Like I had spiders in my stomach not butterflies. (by Unspoken, age 15)

Over the next 20 minutes, Alys talks about her fear and fascination with spiders and ‘toxic’ feelings of ‘attachment’. We Google toxic spiders and read about the difference between toxins and poisons. We find out that ‘venom’ refers to a toxin that is intentionally delivered for the purposes of defence (e.g., in bees). Alys starts to make connections to her silenced clay heart and how, in her words, ‘society is venomous, coming at you’ when you speak out online. We talk about how spiders spin webs and perhaps, the photobook which carries an image of the thorned‐tongue‐trees sparks an idea of making a web from barbed‐wire circling a barbed heart (Figure 5). Alys is keen to draw upon a year's worth of her own instapoetry (over a hundred poems). Across future art lessons, Alys uses the time to select different poems. She chooses one poem for each month of the year (2022–2023). Months later, Alys decides to return to these poems and selects lines that are eventually rolled up and skewered onto the barbs for people to read and interact with, by ‘maybe make their own poetry from’ (Alys). When we share these ideas with Sior, he introduces Alys to Louise Bourgeois' large‐scale steel and bronze spider sculptures. We look at The Tate's internet page on Bourgeois, and the line: ‘I need to make things. The physical interaction with the medium has a curative effect. I need the physical acting out’ (https://www.tate.org.uk/art/artists/louise‐bourgeois‐2351/art‐louise‐bourgeois) stands out. I have returned to this line in the development of this paper, in ways that have led me to explore further how Alys embodies her creative process.

**Figure 5:**
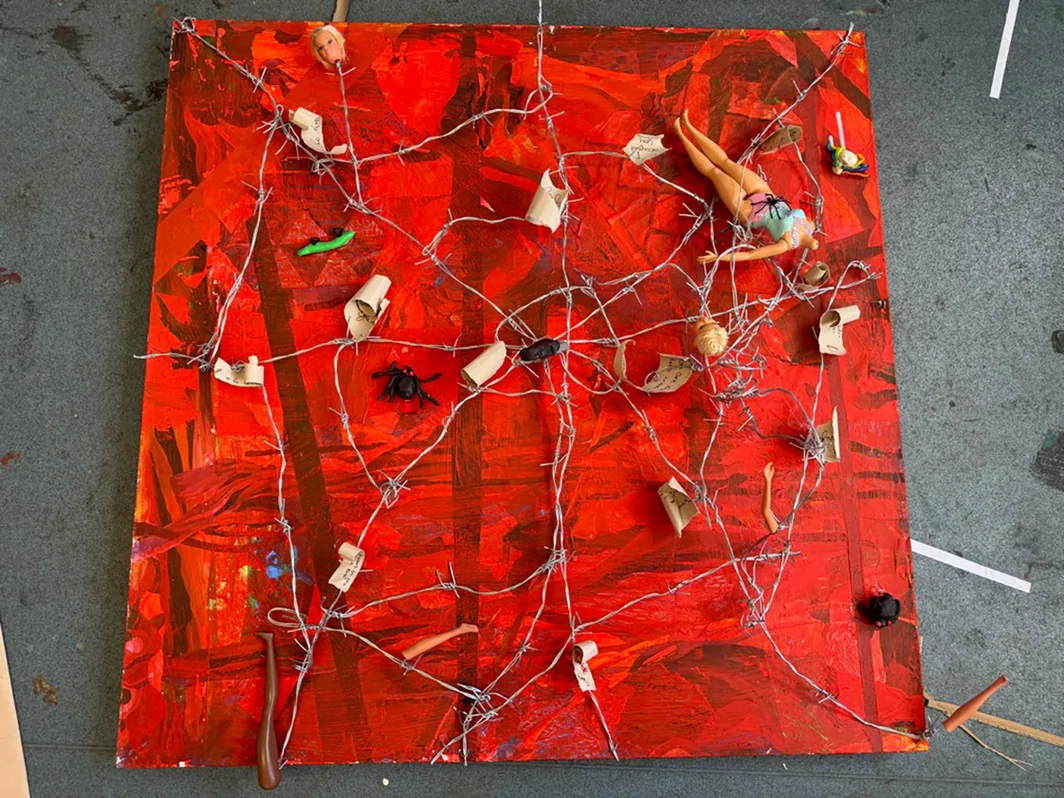
Thorned Web. A large red canvas covered in barbed wire arranged like a spider's web. Skewered onto the barbed web are rolled‐up sticky notes and dismembered body parts from Barbie dolls in various skin tones.

During one of the sessions, where Alys shares some of her poetry, she tells me that she writes her poems in the local woods, ‘down by the river’. Indeed, I began to attune to the affirmative ways in which Alys' creative process has, in the classroom, become more physical, an embodied ontological opening, which she sometimes articulated. For example, in the moulding of the barbed wire with pliers, Alys surprises herself with her own strength, ‘I didn't think I would be able to do it’. When she snaps and skewers Barbie body parts onto her barbed web, she exclaims, ‘this feels so good (laughing)’. She takes delight in rolling up her poetry fragments, sprayed in what Alys humorously calls ‘Lana^9^‐red’, and has fun throwing plastic black spiders onto the web to see where they might land (and which we watch back in slow motion) – perhaps displacing the fear a little and transfixed in watching them (butter)fly. It is a physicality which seems to connect to Alys' own arts‐praxis of surviving the troubles that marble her everyday life. From learning how her poetry is created in place, a further fold, unfolds to other ways in which the history of place matters.

During one of our sessions, I explain how in previous research and engagement projects we have invited young people to bring in photos or objects that matter to them. Alys responds by telling me how she has been sourcing crystals in the local rivers since she was 12 years old – an embodied practice that she later speculates may have come from hanging out with her auntie in a local museum, which includes a crystal exhibit, and her own research (‘I research everything I'm interested in’). A deep sense of knowing surfaces:

Alys: There's this bit where you go under the bridge, that's where I sit (to write poetry). And you go over and then there's like the actual river and there's a bit of stone to the right and basically, like under the ground, there's like actual crystals collected over the years and that's where I get them (…) the first one I found was Aventurine which signifies abundance and then I found Obsidian there and like that signifies protection, the black one. I found like a roll of Obsidian. I was so happy that day. I am quite a big crystal person. I have loads in my room. You can tumble them as well by yourself and I polish them sometimes with my tumbler.

EJ: How do you know … how do you know how to find them?

Alys: You can just tell … like the dirt looks more like moss … the soil is like water … the water is usually clear instead of dirty and like there's a lot of rock, that's usually where you find them.

Raw and tumbled crystals populate her bedroom. She keeps ‘raw crystals’ on her ‘shelf’ and ‘vanity table’, and a rose quartz crystal, also known as ‘the heart stone’, under her pillow. Her ‘favourites’, which she brings to school on a day I missed due to sickness, include ‘Aventurine’, ‘Obsidian’, ‘Rhodonite’, ‘Red Jasper’ and ‘Rose Quartz’. She often carries a selection of crystals in her school bag. When our conversation turns to the ‘Thorned Web’, she points to the barbed heart at the centre of the web and softly whispers that she would like to fill it with a mixture of polished and raw crystals, so that it can be seen and felt in ways that ‘stick out’ from the rest of the web. While the crystals do not appear in the final piece, the process was another moment of shared learning for both of us. This included exploring the book, ‘Crystals in Art: Ancient to Today’ (Haynes and Pissarro 2019) and dropping an image of Daniel Arsham's ‘Blue Calcite Boxing Set’, into her arts‐process powerpoint – an artwork which resonates with Alys's family connections to amateur boxing – and a book we gift to Alys in our final meeting.

Not often do concepts rise so starkly in a project. While we were learning how Alys mined her ecological milieu for crystals that became resourceful posthuman companions to survive and steer her way through some difficult times, we were re‐drawn to Simondon's matterphorical use of the crystal^10^. ‘Crystals are among the most dynamic and seemingly alive products of inorganic nature, the inorganic at the point of its closest meeting with the organic’ (Grosz 2017 p.175). Very crudely, crystals form in response to impurities that enter water. Simondon sees these impurities as problems and the growth of the crystal as a solution to the problem. He describes this process as transduction. While life is clearly not a crystal, the formation of the crystal is perhaps a useful metaphor/matterphor to conceptualise how moving forward and the generation of the new can come from what we already have. We speculate that Alys was already tapping art's political aesthetic potential, not as troubles to overcome, nor as troubles that might be rerouted/changed from within (interiority) or from outside (exteriority), but through an ongoing transindividual, transductive process ‘that is both within us and in the world, which we share with the world’ (Grosz 2017, p. 203). We close this section with a poem and an image, both prompted by an invitation during our final session for Alys to reflect on her participation in the last year of our project (summer 2023 to summer 2024) and, importantly, after Alys had completed all her GCSE coursework and examinations. She selects an image (Figure 6) from among the many additional artworks produced during the unaided 10‐hour GCSE Art examination, which included drawings of spider webs, multi‐coloured clay spiders, papier‐mâché skulls and a cobweb bound ‘confessions jar’. In contrast, the poem below was written and created during this final session:

My heART was bruised

A barbed heart

Imprisoned in my mind

Stripped of my dignity

I can still feel the spiders

But they have stopped biting

The spiders have spared me

For now …

Learning to recognise my feelings

Still mining for crystals

Spinning new webs

New opportunities

To become …

(by Unspoken, age 16)

**Figure 6:**
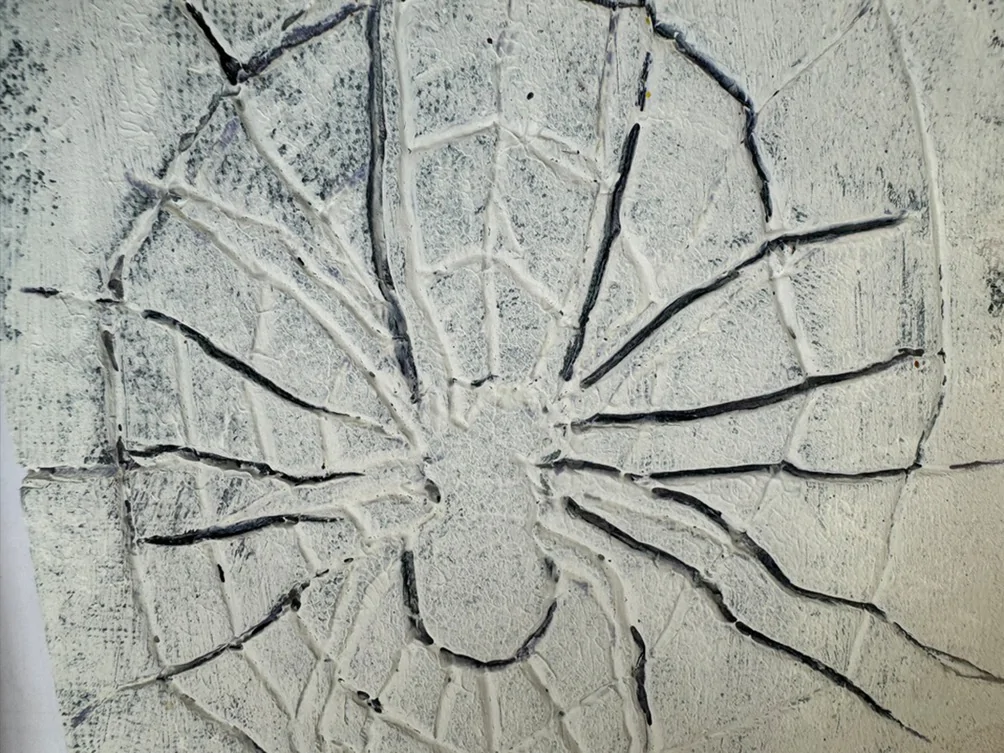
Spinning New Webs. A canvas featuring a slightly raised sculpted spider's web in black and white, with a large spider positioned at the centre, spanning the full width of the web.

## Carrying matters

Dartaphacts, like crystals, have the potential to seed their coordinates in milieus or assemblages that they find themselves in or that we sometimes create. They have the potential to act as posthuman participants in creative co‐produced projects to become vehicles ‘that may touch and set off new becomings’ (Grosz 2017, p. 206). In this final fold we offer three examples that glimpse at the crystallisation of matters sparking a series of potential becomings from the making (process) of the Bruised HeART and Thorned Web: Alys' own creative journey (‘Say it with art’); a digital resource (‘Crystal crush’) and an arts‐based practitioner event (‘Sh(o)ut Out’).

### Say it with art

Sometimes, the making of what matters in arts‐informed research engagement projects not only intra‐acts with young people's own (often hidden) creative worldings, but can also find their way into the formal curriculum^11^. We speculate that on discovering the jouissance of artivism (Nossel 2016) and gaining confidence within a conducive milieu and praxis, the timetabled GCSE art lessons seemed to cleave open an institutional space for Alys to amplify her voice as she integrates her own arts‐praxis (e.g. poetry) with the affective and agitative qualities of working with the visual arts. Moreover, this materialisation of voice amidst all the troubles seemed to open up a previously unimagined future. In the final few months of school, Alys is accepted onto the local college's media studies course. In the final session, she asks again for the link to the personalised Padlet, holding a selection of Alys' anonymised images, sounds and films. She hopes these might enable her to keep creating poems that, ‘turn the pain into something beautiful’, and continue to ‘say it with art’ and voice ‘out loud’ what matters to her, on her journey to ‘change minds’.

### Crystal crush

Becoming response‐able with the materialisation of Wales' new RSE has included co‐producing accessible gender and sexuality education resources and an ongoing RSE professional learning programme (see Renold et al. 2021) to support practitioners in engaging more directly with the human and more‐than human matters on some of the most sensitive folds of experience. One of these resources is the Crush Cards (see www.agendaonline.co.uk/crush, see Renold and Timperley 2023). Over the last 5 years, we have been developing a series of what we call, ‘Crush Cards’. These are an ever‐evolving suite of illustrated data calling cards designed to reanimate research findings and stay close to, and perhaps ‘crush assumptions’ of how children and young people are entangled in, and navigate their way through, complex human and more‐than‐human gender and sexuality assemblages. Each card contains a bold image on one side and research data in the form of direct quotes from children and young people or ethnographic narratives on the other. The images are striking and varied. Crucially, they are mostly of objects not people. So immediately, you enter the field of RSE via the non‐human. The next iteration of the Crush Cards will include Alys' case study and other case studies from the *Unboxing RSE* project. One side will include an image of a rose quartz crystal. On the other, the unfolding vignette of the making of the Bruised Heart. This vignette will story some of Alys' inventive and creative practices of living with and surviving past and contemporary troubles. At the bottom of the card, there will be a link to this paper, as part of our wider project to make research evidence matter differently. We anticipate that future Crush Card participants might connect the image to the current crystal craze (#crystaltok) among young people in recent years (Wilkinson 2022), but in overturning, might be rerouted to the resourcefulness of Alys mining her own crystals for peace, courage, abundance and protection. And perhaps, for some, a more ‘creative ecology’ (Harris 2016, 2021; Harris and Holman Jones 2022) for re‐image‐ining RSE might be seeded.

### Sh(o)ut out

In the summer of 2024, we design and facilitate a one‐day practitioner workshop with artists, practitioners and academics. Inspired by Alys' words we called the event ‘If you can't say it out loud, say it with art: Getting creative with RSE provision’. The event builds on over 5 years of co‐producing resources and delivering an extensive professional learning programme and series of events (see https://agendaonline.co.uk/crush‐2024/) all designed to foreground the ways we might ‘attune’ to and open up ‘what matters’ so that the curriculum can be ‘relevant and responsive’ to what ‘children and young people are encountering in society’ (Welsh Government 2022a, 2022b). For the purposes of this paper, we offer a glimpse into how the ‘affective tonality’ (Massumi 2011) of the materialisation of youth voice came to matter, through a short vignette below:

The morning session opens with the film, ‘Creative Curious and Cross‐Curricular RSE’ (see https://vimeo.com/910473537). This film shares moments from a range of different research and engagement projects with teachers and artists, including the making of the sculpture and film Bruised Heart, among other dartaphacts from the Unboxing RSE project. As the day unfolds, we open one of the boxes, inviting participants to story ‘what matters’ to young people using a range of images from the Crush Card resource. Later, we invite them to sculpt with ‘what matters.’ Here, participants are invited to open a second box, which contains the ingredients for making salt dough figures. Included are some of the materials that surfaced in the Unboxing RSE dartaphacts, including a handful of screws from the Bruised HeART. Over the next half hour a mix of abstract and recognisable shapes, bodies and body parts assemble, including a mouth with a large protruding tongue, next to a speech bubble with the words ‘have your say’ carved in it (Figure 7), a pair of plump lips clamped shut with three metal screws (Figure 8) and a large heart with the word, ‘Empathy’ shaped with netting in its centre, surrounded by five green, purple and pink feathers, and framed by a raised crust and pierced with multiple holes (Figure 9).

**Figure 7:**
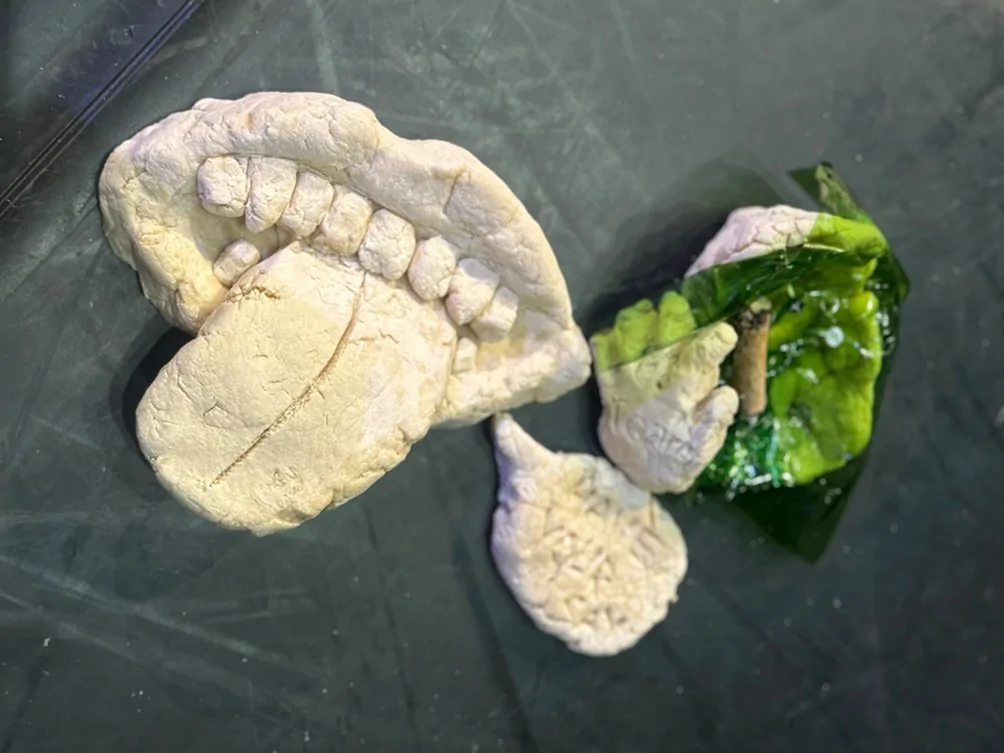
Have Your Say. A salt‐dough sculpture of a mouth with visible teeth and a protruding tongue. Beside it, there's a clay speech bubble with the words ‘have your say’ carved into it, and a clay hand with the word ‘learn’ carved onto the back of the hand.

**Figure 8:**
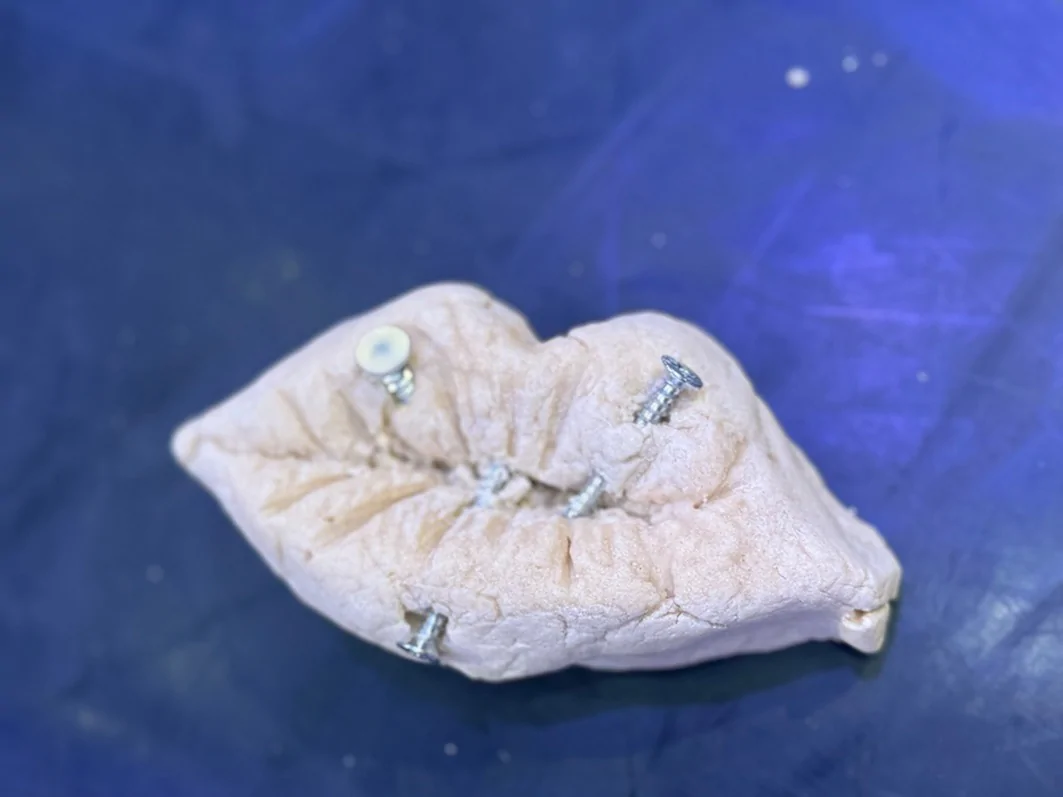
Screwed Lips. A small salt‐dough sculpture of lips clamped shut with three metal screws.

**Figure 9:**
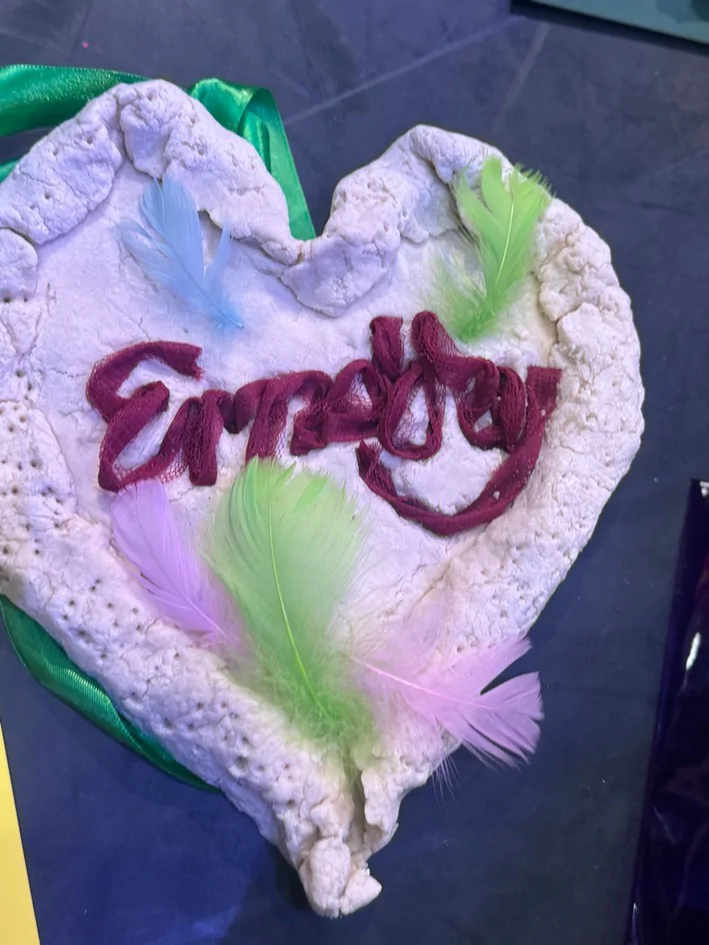
Empathy HeART. A flat salt‐dough heart with a raised outline pierced by tiny holes. The word ‘empathy’ is formed from dark red  at the centre, surrounded by a selection of light pink, green, and blue feathers.

We speculate that the affective‐material tonality of the Bruised HeART is folded into and charges the practitioners' process. The making of voice continues to surface throughout the day, and the power of creative listening jostles with the tension of how we might make safe spaces to unbox the silence that permeates so much RSE provision, for practitioners and pupils.

## Open ending: keeping matters on the move, and on the make

This paper is in dialogue with an expanding body of new‐materialist posthuman research praxis that is increasingly leveraging the affective power of visual arts to surface what might be mattering to young people in the field of relationships and sexuality education (see Strom *et al*. 2019; Allen 2021; Lupton and Leahy 2021; Renold *et al*. 2025). Examples of some of this creative co‐production include speculative filmmaking and storytelling (Timperley 2020; Pasley 2020; Wolfe 2024); craftivist collaging (Ringrose *et al*. 2021; Zarabadi 2021); hashtag activist workshops (Pihkala and Huuki 2019); making wax ‘fruiting bodies’ (Marston 2025); and life drawings (Stanhope 2022). What binds these new‐materialist projects is how the experiential artefacts (or ‘dartaphacts’) created, have *taken form* to *in‐form* and *trans‐form* diverse publics, practices and policies. These projects do not approach the onto‐epistemological ‘turn’ as detached observers. Instead, they embody what Stengers (2021, p. 89) describes as ‘engagement all the way down’, enacting activist practices and choreographing political engagement with stakeholders for whom these practices matter deeply. Indeed, this body of work increasingly highlights the ethical and political dimensions of ‘becoming response‐able’ (Barad 2007) in crafting and communicating projects that foreground and interrogate gender and/or sexuality across supportive and challenging socio‐political contexts.

In contrast to many of the projects above, the arts‐praxis shared in this paper has been developing for over a decade of working in the same post‐industrial place, and with the same artists (see Renold and Ivinson forthcoming). Over time, we have learned how to attune to the human and more‐than‐human folds, forces and feelings that sometimes go unrecognised by researchers and practitioners working with young people. We have learned to become more and more attuned to how ‘what matters’ for young people living in post‐industrial locales can surface in multi‐sensory arts‐informed projects as ways of surviving, staying and making with community troubles that are always more than theirs.

In previous writing we have argued how making dartaphacts with young people can be where ‘things in the making cut their transformational teeth’ (Massumi 2015 ix). Our approach is to attune to these potential future passageways and imaginative leaps through the making of dartaphacts – micro‐political enunciators crafted from and carrying what might be mattering.

Consider how the Bruised HeART was formed in the act of making, once a conducive milieu was felt and leaned into (e.g. transforming early ideas to make a cute LGBTQ rainbow heart into a snipped, screwed and slashed heart‐tongue). Dartaphacts, like the Bruised HeART, are thus not creative outputs representing what matters. Rather, they are non‐representational, relational, lively matter – living beings generated in a co‐productive praxis of coming ‘to know something by yielding to it and following where it goes’ (Marks 2024, 30) within and beyond an arts classroom. In this paper, we have mapped this journey across a series of three figurative folds, in a rare case study that has allowed us to glimpse at the unfolding/enfolding ethical–political aesthetics of multiple ‘cries that matter’ (Stengers 2021). In *Crushing Matters* we witnessed episodes of silence and speaking out braiding across time‐zones. Here, it became possible to detect a folded creative feminist legacy of local and global knowing, learning and change‐making. In *Crystalising Matters* we saw Alys' embodied and emplaced creative inventiveness tap into the auto‐poesies intra‐action between words and minerals, in ways that seem to generate a forward force to navigate contemporary troubles – a ‘queer r/age’ steeped in and subverting societal assumptions of ‘too young to know’. Across each fold, we have tried to illustrate how folded dartaphacts can sometimes unfold in situ and immediately register (such as Alys' interjection of her family connection to the Aberfan disaster). And sometimes, these enfoldings/unfoldings have surfaced as uncanny and haunting moments, sending affective shock waves and wanderlines through a praxis (such as Alys' poem, which discursively depicts almost identical images of pierced hearts in a film yet to be made – thorny matters that Alys continued to explore in her GCSE artwork with the barbed‐wire web). In the final fold, *Creating Matters*, we offered examples of how dartaphacts as posthuman participants have become and continue to become micro‐political enunciators, transporting traces of what matters into new places, spaces and publics: for Alys (perhaps into a new media studies A level course) for practitioners (perhaps on their journey to develop a co‐constructed creative, curious and cross‐curricular RSE) and for future RSE resources designed specifically to unfold assumptions of what young people might know, feel and act, and in ways that might cultivate possibilities for a more meaningful and engaging RSE.

While these figurations are only partial insights into a folding process, it is possible to recognise the creative emergence of ‘new folds constantly being pulled from existing thoughts, histories and materials in unique local situations’ (Marks 2024 p. 30/31). This process does not impose, assume, or dictate the creative methods, or ‘what matters’ in the creative process from above, outside, or at a distance. Rather, it recognises how experience is always already inventive and how attuning to the more sensitive folds of experience, demands a careful, ethical and political praxis of making‐with, and sometimes becoming more‐than the troubles already in motion. This is a form of co‐production that works creatively with the vitality of what matters, with young people, from the ground up, from within the/ir world, and then curating and communicating *what matters* within contexts that might continue to enfold and unfold, and spark new ways of understanding and doing relationships and sexuality education Otherwise.

## Endnotes

1 This project is a Wellcome Trust funded public engagement research project, ‘Engaging Sexual Stores’ (Reference UNS100842) and conducted in collaboration with The Open University, UCL London, Cardiff University, and Brook (national sexual health charity). See https://www.natsal.ac.uk/related‐projects/collaborations/engaging‐sexual‐stories/ for further details. The project received ethical approval from Cardiff University. While each young person and their parent/carer signed a consent form before taking part in the research, the process of informed consent was on‐going throughout the fieldwork sessions (see Renold al. 2008).

2 While this paper is solo‐authored, I mostly use the collective noun, ‘we’ throughout to acknowledge the collaborative praxis of slow coproduction developed with Professor Gabrielle Ivinson for over 10 years in this post‐industrial place. Members of the team for the UnboXing RSE project included academics Dr. Victoria Timperley and artists, Heloise Godfrey‐Talbot and Rowan Talbot.

3 This first session included an activity which invited young people to write on clouds and/or sealed envelopes what they thought would make the space safe and supportive for them explore and share potentially sensitive issues (see the section on ‘safety and support clouds’ in Renold & Timperley 2024).

4 We use the term ‘intra‐view’ rather than ‘interview’ to acknowledge our relational entanglement in the making process, and our speculative aim to generate new ‘matters’ (e.g. questions, ideas, feelings) in re‐viewing the films with young people on the process of becoming a participant in the project.

5 In a longitudinal project it is possible to make space and time for the processual nature of what we have theorised elsewhere as ‘becoming participant’ (Renold et al. 2008). In doing so, we are also able to demystify and make transparent our iterative praxis as para‐academics (see Renold and Ivinson 2022; Renold & Ringrose 2025). With Alys, I could regularly discuss what and how words and images can become research data, how I was making sense of some of her journey. This included crafting and gifting back an analytic data‐poem, an aesthetic mode that mirrored Alys' own creative meaning‐making processes. Many of these discussions included the importance and process of anonymisation. Alys has given permission for all the anonymised quotes, poems and images in this paper to be included. Some details have been changed or remain opaque to ensure anonymity.

6 The slash in r/age is to punctuate the ways in which developmental discourses often work to undermine the intense feelings of anger expressed by preteens about their own and wider gender and sexual injustices in the world.

7 Each young person, or group, created an artist's tag for their artwork. Alys' tag, was ‘unspoken’.

8 GCSE stands for General Certificate of Secondary Education. It is a qualification taken by most students in England, Wales, and Northern Ireland at the end of their compulsory secondary school education (typically at age 16).

9 ‘Lana‐red’ is a reference to the American singer‐songwriter Lana Del Rey.

10 Huge acknowledgement here to Professor Gabrielle Ivinson in drawing out the crystalising matterphor, which we are developing more fully in our forthcoming book, Creative Coproduction with Young People: A postqualitative pARTicipatory praxis' (Renold & Ivinson forthcoming).

11 We hope to write about the entangled ethical‐political‐pedagogic relations of coproducing dartaphacts which inspire and fold into the formal curriculum, such as GCSE Art, in future publications.
